# Histological Evidence for the Enteric Nervous System and the Choroid Plexus as Alternative Routes of Neuroinvasion by SARS-CoV2

**DOI:** 10.3389/fnana.2020.596439

**Published:** 2020-10-06

**Authors:** Felix Deffner, Melanie Scharr, Stefanie Klingenstein, Moritz Klingenstein, Alfio Milazzo, Simon Scherer, Andreas Wagner, Bernhard Hirt, Andreas F. Mack, Peter H. Neckel

**Affiliations:** ^1^Institute of Clinical Anatomy and Cell Analysis, University of Tübingen, Tübingen, Germany; ^2^Institute of Neuroanatomy and Developmental Biology, University of Tübingen, Tübingen, Germany; ^3^Department of Pediatric Surgery, University Children’s Hospital, Tübingen, Germany

**Keywords:** SARS-CoV2, neuro-COVID, neuroinvasion, enteric nervous system, choroid plexus

## Abstract

Evidence is mounting that the novel corona virus SARS-CoV2 inflicts neurological symptoms in a subgroup of COVID-19 patients. While plenty of theories on the route of neuroinvasion have been proposed, little histological evidence has been presented supporting any of these hypotheses. Therefore, we carried out immunostainings for ACE2 and TMPRSS2, two proteinases crucial for the entry of SARS-CoV2 into host cells, in the human enteric nervous system (ENS), as well as in the choroid plexus of the lateral ventricles. Both of these sites are important, yet often neglected entry gates to the nervous system. We found that ACE2 and TMPRSS2 are expressed by enteric neurons and glial cells of the small and large intestine, as well as choroid plexus epithelial cells, indicating that these cells meet the molecular requirements for viral entry. Together, our results are fundamental histological evidence substantiating current theories of neuroinvasion by SARS-CoV2.

## Introduction

Since December 2019, the current COVID-19 pandemic, caused by the severe acute respiratory syndrome coronavirus 2 (SARS-CoV2), led to over 21.2 million reported cases and more than 761,700 deaths around the globe (WHO, 2020). Symptoms primarily involve coughing and dyspnea, but also fever, muscle soreness, acute respiratory distress syndrome, or diarrhea (Sun et al., 2020). While the respiratory symptoms most often are decisive for intensive care measures (Wang et al., 2020), approximately 30% of COVID-19 patients suffer from additional neurological symptoms including anosmia, dysgeusia, headache, fatigue, neuralgia, disorientation, epileptic seizures, pyramidal signs, nausea, or vomiting (Bösel and Berlit, 2020; Leonardi et al., 2020). Therefore, interest sparked in the pathogenesis of these neurological manifestations and more urgently in the routes of neuroinvasion by SARS-CoV2. While the neuroinvasive routes via the olfactory epithelium/olfactory nerve or via the blood-brain barrier have gained much attention, viral transmission via the enteric nervous system (ENS) or the cerebrospinal fluid (CSF), remains poorly investigated.

Recently, the angiotensin I converting enzyme 2 (ACE2) was shown to serve as a cellular receptor on the host cell membrane essential for transmission by SARS-CoV2 (Qiu et al., 2020; Yan et al., 2020; Zhou et al., 2020). The exact mechanisms of virus entry remain fragmentary, but binding of the viral spike protein (S protein) to ACE2 as well as priming of S proteins by the serine protease TMPRSS2 are substantial elements of the process (Hoffmann et al., 2020). Surprisingly, the expression patterns of ACE2 and TMPRSS2, which determine cellular entry routes, have been investigated scarcely in human organs other than the respiratory system (Ziegler et al., 2020). While previous studies found ACE2-mRNA expression in virtually all human organs (Devaux et al., 2020), the corresponding protein expression was much more restricted to epithelia, especially in enterocytes of the small intestine, alveolar epithelial cells of the lungs, vascular endothelial cells, proximal tubular cells, and glomerular epithelium in the kidney, but also, less intense, in smooth muscle cells (Hamming et al., 2004).

Since gastrointestinal symptoms and the presence of virus nucleic acids in the stool of COVID-19 patients have been reported repeatedly (Li Z. et al., 2020; Zhang et al., 2020), Esposito et al. (2020) recently suggested that the ENS could serve as an entry route to the brain. One of the proposed pathways implicate that infected enteric neurons could serve as direct entry to the CNS by passing on the virus via the gut brain axis (e.g., via the vagus nerve or splanchnic nerves). In fact, a comparable mechanism with infection of enteric neurons with successive virus persistence (Brun et al., 2010) as well as neurogenic transmission was previously shown for herpes (Khoury-Hanold et al., 2016) and influenza (Park et al., 2002) viruses. Additionally, Esposito et al. (2020) propose a secondary effect, by which an infected ENS could contribute to and aggravate the cytokine storm elicited by COVID-19. This theory is substantiated by extensive work showing the immunological properties of enteric glial cells (reviewed in detail by Yoo and Mazmanian, 2017). Yet, although other coronaviruses, such as SARS-CoV and MERS-CoV, cause gastrointestinal symptoms as well (Petrosillo et al., 2020), a detailed investigation of the protein expression of ACE2 or TMPRSS2 has not been carried out in the ENS so far.

Various coronaviruses were shown to enter the cerebrospinal fluid (CSF) both in animal experiments and in patients with neurological symptoms (De Felice et al., 2020). In addition, RNA of the SARS-CoV and of the current SARS-CoV2 were detected in the CSF of patients with neurological symptoms (Hung et al., 2003; Moriguchi et al., 2020). Yet, little is known about how these viruses enter the CSF (Cataldi et al., 2020). While most authors proposed a dysfunctional blood–brain barrier (De Felice et al., 2020), the anatomical route via fenestrated capillaries in the circumventricular organs and the choroid plexus, and successively the potential infection of ependymal and plexus epithelium cells has been largely neglected. Moreover, contrary to the exclusively neuronal transmission through the *lamina cribrosa* mentioned above, coronaviruses were also proposed to enter the CSF via the nasal mucosa and subsequently the perineural spaces of the olfactory nerve (Li Z. et al., 2020), thereby allowing for a secondary infection of circumventricular areas. Therefore, it is conceivable that virus particles enter the brain parenchyma at these locations, potentially leading to dysregulation of the water-electrolyte homeostasis in the CSF and brain or even act directly on neuronal functions of circumventricular circuits, such as in the *area postrema*. Both of these mechanisms could explain reported symptoms of dizziness or vomiting in COVID-19 patients. Although similar infection routes are known from other pathogens, no data on the protein expression and localization of ACE2 and TMPRSS2 is available in these areas of the human brain.

In this work, we focused on the protein expression and localization of ACE2 and TMPRSS2 in tissues at the interface between the nervous system and non-neuronal organs and systems. To do so, we carried out immunostainings on paraffin and cryosections of the small and large intestine, choroid plexus, and adjacent brain parenchyma in patient-derived and *post-mortem* material. We report evidence for the expression of ACE2 and TMPRSS2 on protein level in the ENS, choroid plexus epithelium, and the blood brain barrier, which previously only has been presumed based on mRNA data. Thus, our data are constitutive histological evidence for hypothesized alternative routes for neuroinvasion by SARS-CoV-2.

## Materials and Methods

### Patient Specimens

Human gut samples were obtained from nine males and female patients aged 3 months and 8 years who were operated due to imperforate anus, intestinal obstruction syndrome, or rhabdomyosarcoma (Supplementary Table S1). All samples were collected after approval of the local ethical committee (Project Nr. 652/2019BO2) and with the consent of the patients’ parents.

### *Post-mortem* Specimens

Human choroid plexus was collected from eight cadavers donated to the Institute of Clinical Anatomy and Cell Analysis in Tübingen by female and male volunteers aged between 74 and 94. The body donors gave their informed consent in concert with the declaration of Helsinki to use the cadaver for research purposes. The procedure was approved by the ethics commission at the Medical Department of the University of Tübingen (Project Nr. 237/2007 BO1). Samples were taken within 8 to 19 h *post-mortem* (Supplementary Table S2).

### Histological Workup

Before embedding, tissue samples were fixated with 4% (w/v) phosphate buffered p-formaldehyde (Applichem, Darmstadt, Germany) overnight and rinsed three times with phosphate-buffered saline (PBS).

For cryoconservation, fixed samples were frozen in isopentane-nitrogen cooled TissueTek^®^ (Sakura, Staufen, Germany) and stored at −80°C until further processing. Before staining, cryosections (15 μm) were dried for 1 h at room temperature, following rehydration with distilled water for 30 min.

For paraffin embedding, fixed tissue samples were dehydrated in an ascending alcohol series, followed by xylene and overnight infiltration of Paraffin at 60°C. Before staining, paraffin sections (5–10 μm) were dewaxed by xylene and a descending alcohol series and were rinsed once with distilled water. Next, sections were pre-treated with boiled citric acid monohydrate buffer (10 mM, pH 6.0, Merck, Darmstadt, Germany) for 3 min and cooled down at room temperature.

### Immunohistochemistry

To prevent unspecific binding of antibodies, samples were blocked for 30 min with PBS containing 4% (v/v) goat serum (Biochrom, Berlin, Germany), 0.1% (v/v) bovine serum albumin (Roth, Karlsruhe, Germany), and 0.1% (v/v) Triton^®^ X-100 (Roth, Karlsruhe, Germany), followed by incubation of primary antibodies (Supplementary Table S3) diluted in PBS with 0.1% (v/v) bovine serum albumin and 0.1% (v/v) Triton^®^ X-100 overnight at 4 °C in a humidity chamber. Afterward, samples were washed with PBS three times for 5 to 10 min. The secondary antibody (Supplementary Table S4) was diluted in PBS, 0.1% (v/v) Triton X-100, and 0.1% (w/v) BSA and incubated for 60 to 90 min at room temperature. Nuclear staining was carried out with 4′,6-diamidino-2-phenylindole (DAPI) or DRAQ5 solution (200 ng/ml; Roth). After two washing steps with PBS for 5 to 10 min, the samples were washed in distilled water for 5 min, followed by mounting with Kaiser’s glycerol gelatine (Merck, Darmstadt, Germany) or Mowiol 4-88 (Roth).

### Microscopy

Images were acquired using a Zeiss Axio Imager.Z1 fluorescence microscope (Zeiss, Jena, Germany) with Apotome module, as well as a confocal LSM510 Meta with laser lines at 488, 543, 633 nm for excitation and appropriate filter sets. Images were acquired using ZEN software (Zeiss).

## Results

### ACE2 and TMPRSS2 in the Human Intestine

**FIGURE 1:**
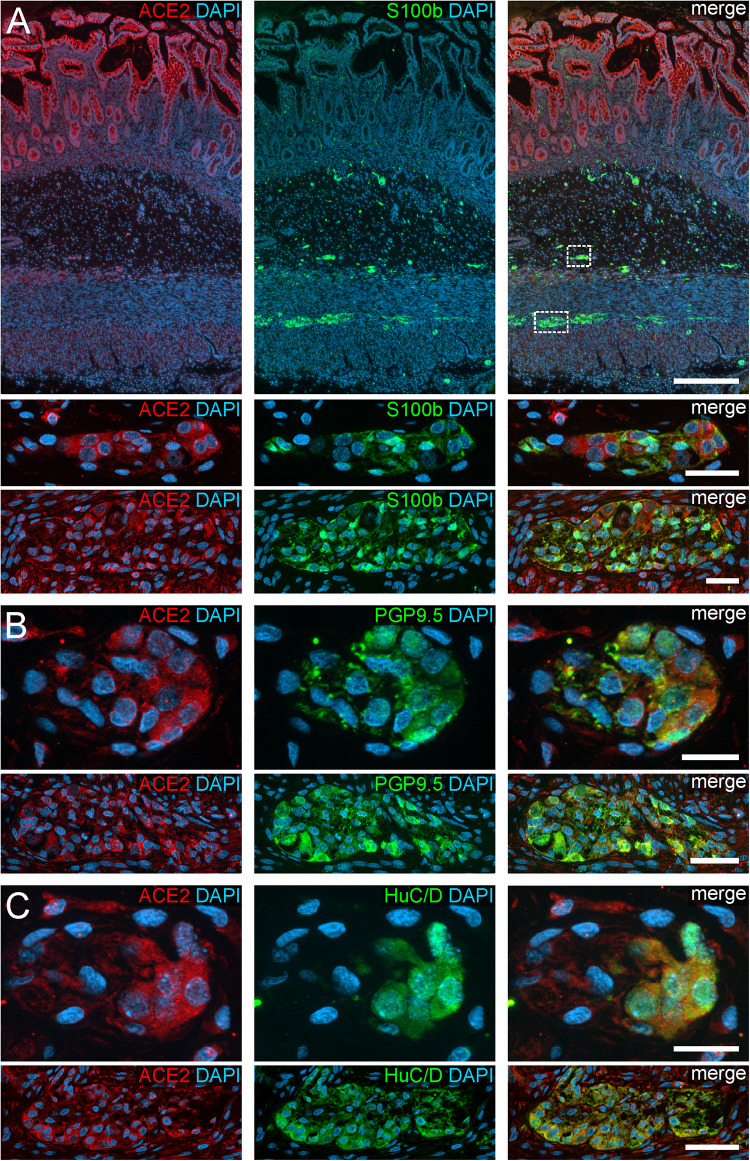
ACE2 expression in the human ENS of the small intestine. **(A)** Overview of the entire gut wall of a small intestinal segment with immunofluorescence stainings for ACE2 (red), the glial marker S100b (green), and with the nuclear marker DAPI (blue). The white rectangles indicate the location of the high power magnification micrographs below showing a representative submucous and myenteric ganglion. **(B,C)** show representative submucous and myenteric ganglia stained for ACE2 (red), DAPI (blue), and the neuronal markers PGP9.5 (**B**, red) or HuC/D (**C**, red). Clearly, a positive staining can be found in enteric neurons and, less intense, in glial cells. The overview is a standard epifluorescence image; details are maximum intensity projections of optical sections by structured illumination. Scale bars: overview 250 μm; details 50 μm.

**FIGURE 2:**
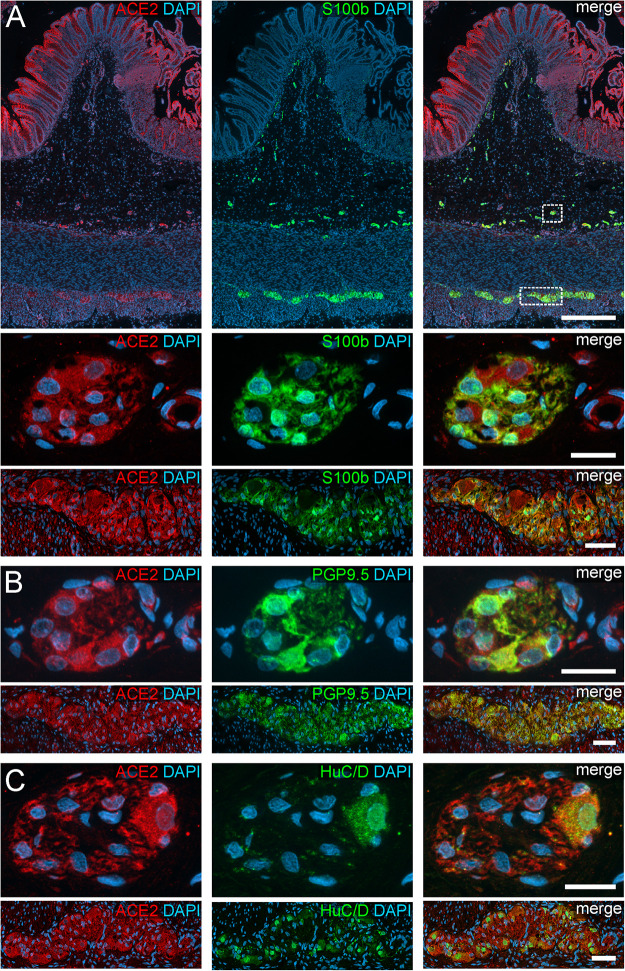
ACE2 expression in the human ENS of the large intestine. **(A)** Overview of the entire gut wall of a colon segment with immunofluorescence stainings for ACE2 (red), the glial marker S100b (green), and with the nuclear marker DAPI (blue). The white rectangles indicate the location of the high power magnification micrographs below showing a representative submucous and myenteric ganglion. **(B,C)** show representative submucous and myenteric ganglia stained for ACE2 (red), DAPI (blue), and the neuronal markers PGP9.5 (**B**, red) or HuC/D (**C**, red). The ACE2 staining can be found in neurons and glial cells and is considerably stronger in the colon compared to the small intestine. The overview is a standard epifluorescence image; details are maximum intensity projections of optical sections by structured illumination. Scale bars: overview 250 μm; details 50 μm.

**FIGURE 3:**
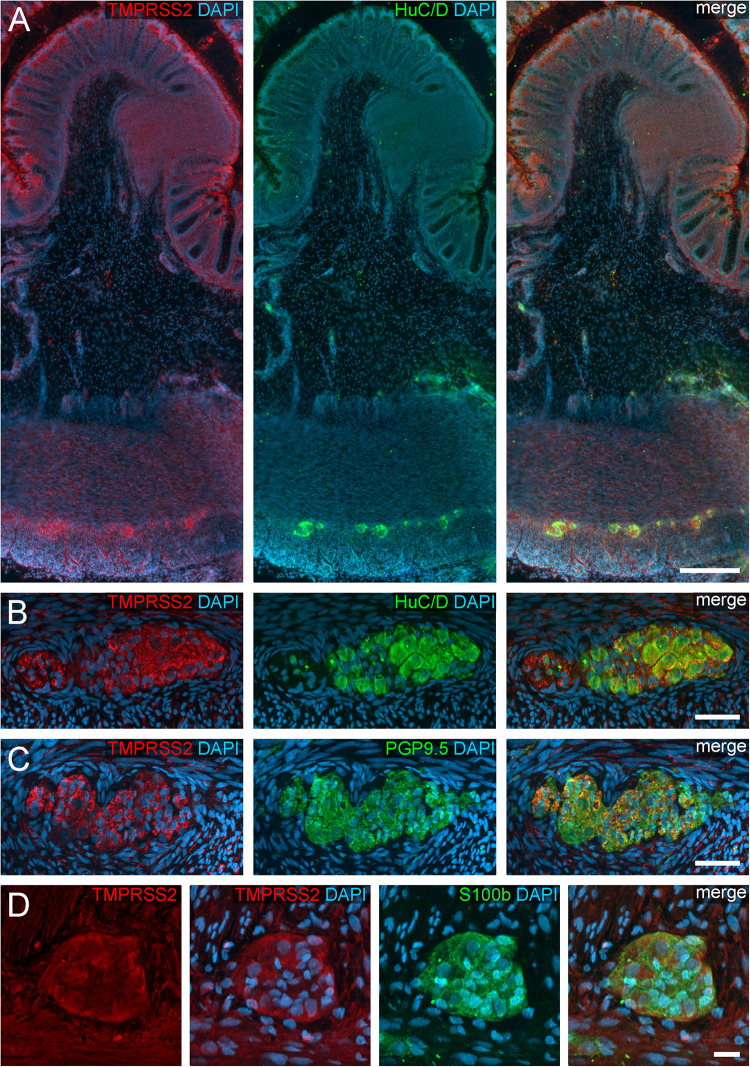
TMPRSS2 expression in the human ENS. **(A)** Overview of the entire gut wall of a colon segment with immunofluorescence stainings for TMPRSS2 (red), the neuronal marker HuC/D (green), and the nuclear marker DAPI (blue). **(B,C)** show representative large intestinal myenteric ganglia stained for TMPRSS2 (red), DAPI (blue), and the neuronal markers HuC/D (**B**, red) or PGP9.5 (**C**, red). **(D)** Representative myenteric ganglion in the small intestine stained for TMPRSS2 (red), the glial marker S100b (green), and the nuclear marker DAPI (blue). Note that TMPRSS2 stainings were markedly stronger in enteric ganglia in the colon **(A–C)** than in the small intestine **(D)**. The overview is a standard epifluorescence image; details are maximum intensity projections of optical sections by structured illumination. Scale bars: **(A)** 250 μm; **(B–D)** 50 μm.

Immunoreactivity for ACE2 and TMPRSS2 was detected in various layers and tissues of the intestinal wall (Figures 1–3), including the ENS (detailed in the next section). Our stainings confirmed previously reported expression of ACE2 in the epithelial lining of the small and large intestine (Supplementary Figure S2). While the expression was especially strong in the brush border of the small intestine, there was also a cytoplasmic staining clearly distinguishable. In addition to previous findings, we found TMPRSS2 expression in the cytoplasm of enteric epithelial cells, both in the small and large intestine. Although we occasionally found a strong fluorescence at the apical cell boundary, we did not detect a clear localization of TMPRSS2 in the microvilli as we did for ACE2 (data not shown).

Furthermore, we found ACE2 expression in smooth muscle cells in the *Tunica muscularis* as well as in the muscular *Tunica media* of arterioles within the intestinal wall. These smooth muscle cells also exhibited a uniform cytoplasmic localization of ACE2 without visible concentration of ACE2 at the plasma membrane. Moreover, we detected TMPRSS2 in the *Tunica muscularis* as well, yet considerably weaker than in the epithelium. Occasionally, endothelial cells of small capillaries especially in the *Tela submucosa* exhibited a uniform, cytoplasmic staining for ACE2. Although the endothelium of larger blood vessels was negative, we found ACE2 staining of the smooth musculature in the *Tunica media* of these vessels. TMPRSS2, however, was not expressed by endothelial cells of the small and large intestine.

### ACE2 and TMPRSS2 in the Enteric Nervous System

In addition to the expression pattern in the intestinal epithelium in previous reports, we payed special attention to the localization of ACE2 and TMPRSS2 in the ENS. Interestingly, we found a cytoplasmic expression of ACE2 in the perikarya of enteric neurons as well as glial cells, both in the myenteric and submucous plexus (Figures 1, 2). The fluorescent intensity of neuronal ACE2 was weaker in myenteric neurons than in submucosal nerve cells. Intriguingly, however, the staining of glial cells tended to be stronger in the myenteric ganglia compared to submucosal ganglia. Moreover, the myenteric plexus of the small intestine was stained less intense for ACE2 than the myenteric ganglia of the large intestine. Although the staining of the myenteric ganglia was clearly distinguishable from the surrounding ACE2-positive smooth musculature in all investigated cases, the fluorescent intensity of most neural cells was lower when compared to the intensely stained brush border of the small intestinal enterocytes. Interestingly, we found that enteric neurons exhibited different levels of ACE2 staining intensity (Supplementary Figure S3), suggesting that the expression level of ACE2 may vary between neuronal subtypes. It is also noteworthy that we did not detect any staining for ACE2 in the large neuronal fiber bundles outside the ganglia or in the fine neurite network surrounding the mucosal crypts, strongly indicating that ACE2 is not expressed in the dendrites or axons of enteric neurons or extrinsic nerve fibers. Moreover, we did not find any ACE2 expression in glial cells residing in the *Lamina propria mucosae* (type-III enteric glial cells) in the small and large intestine (Supplementary Figures S4A,B). In contrast, most extraganglionic enteric glia in the submucosa stained for ACE2 (Supplementary Figure S4C), although considerably weaker than type-I and type-II glial cells within the ganglia and the connectives. Interestingly, we also found enteric glial cells making contact to smaller blood vessels with varying ACE2 staining intensities (Supplementary Figure S4D). Within the *Tunica muscularis*, type-IV enteric glial cells also expressed ACE2, however, this staining was equally intense as the surrounding tissue (Supplementary Figures S4E,F).

We also found TMPRSS2 expression in the ganglia of the myenteric and submucous plexus (Figure 3). Both, enteric neurons and glial cells exhibited cytoplasmic staining, partly with marbled patterns or with fluorescent punctae. Intriguingly, the overall fluorescent intensity of enteric ganglia was noticeably stronger in the colon compared to the small intestine. Thus, small intestinal myenteric ganglia often stained equally intense as the surrounding smooth muscle tissue (Figure 3D and see Supplementary Figure S1 for negative controls), whereas ganglia in the large intestine exhibited a highly fluorescent TMPRSS2 staining. It is therefore conceivable, that the expression of both proteases differs between the ganglia of different intestinal segments. Moreover, we did not detect a TMPRSS2 signal in the exreganglionic (type-III) glial cells in the Tela submucosa and Lamina propria mucosae (data not shown).

### ACE2 and TMPRSS2 in the Human Choroid Plexus

**FIGURE 4:**
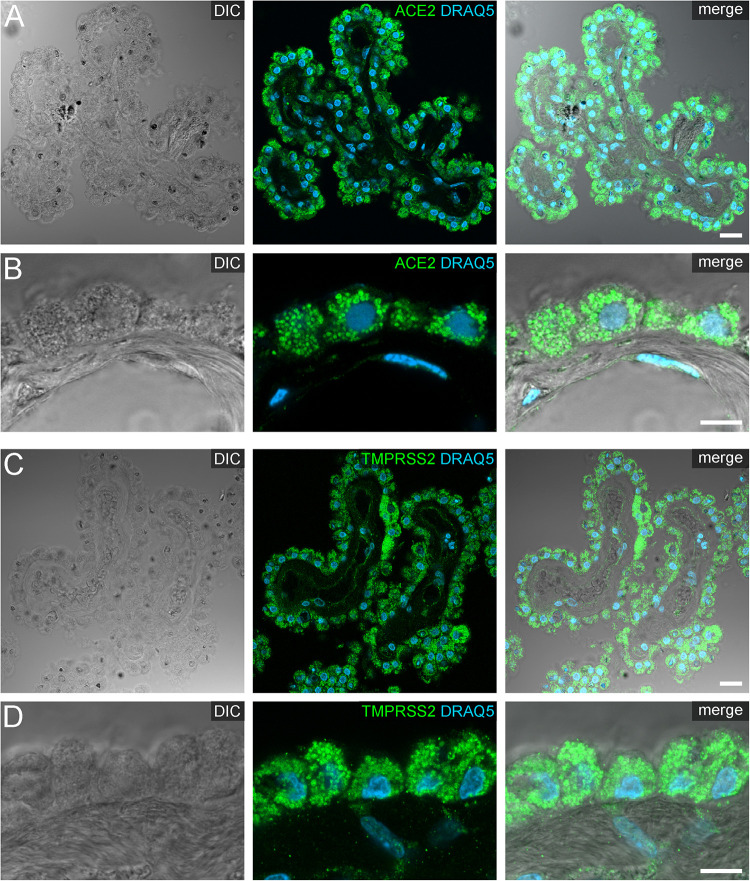
Expression of ACE2 and TMPRSS2 in the human choroid plexus. **(A)** Overview and **(B)** high power images of sections through the human choroid plexus of the lateral ventricle, in a transmitted light DIC image (left), immunostained for ACE2 (green) and the nuclear marker DRAQ5 (blue; middle). **(B)** Shows representative plexus epithelial cells clearly positive for ACE2 (green). **(C)** Overview and **(D)** high power images of sections through the human choroid plexus of the lateral ventricle immunostained for TMPRSS2 (green) and the nuclear marker DRAQ5 (blue; middle). **(D)** Shows that TMPRSS2 has a similar distribution in choroid plexus epithelial as ACE2. All images are single optical sections (pinhole size 1 AU). Scale bars: **(A,C)** 20 μm; **(B,D)** 10 μm.

Our staining on post-mortem human choroid plexus revealed an intense protein expression of ACE2 and TMPRSS2 in the vast majority of plexus epithelial cells (Figure 4). Both proteases were localized predominantly in intracellular granules, arguably representing lysosomes or large vesicles. Occasionally, we detected ACE2 immunoreactivity at the apical and/or basolateral cell surface of epithelial cells (Supplementary Figure S5). Generally, the staining intensity was heterogeneous, especially in sections stained for TMPRSS2 we found few cells scattered throughout the epithelium with a considerably higher expression throughout the entire cytoplasm when compared to neighboring epithelial cells (Figure 4C).

**FIGURE 5:**
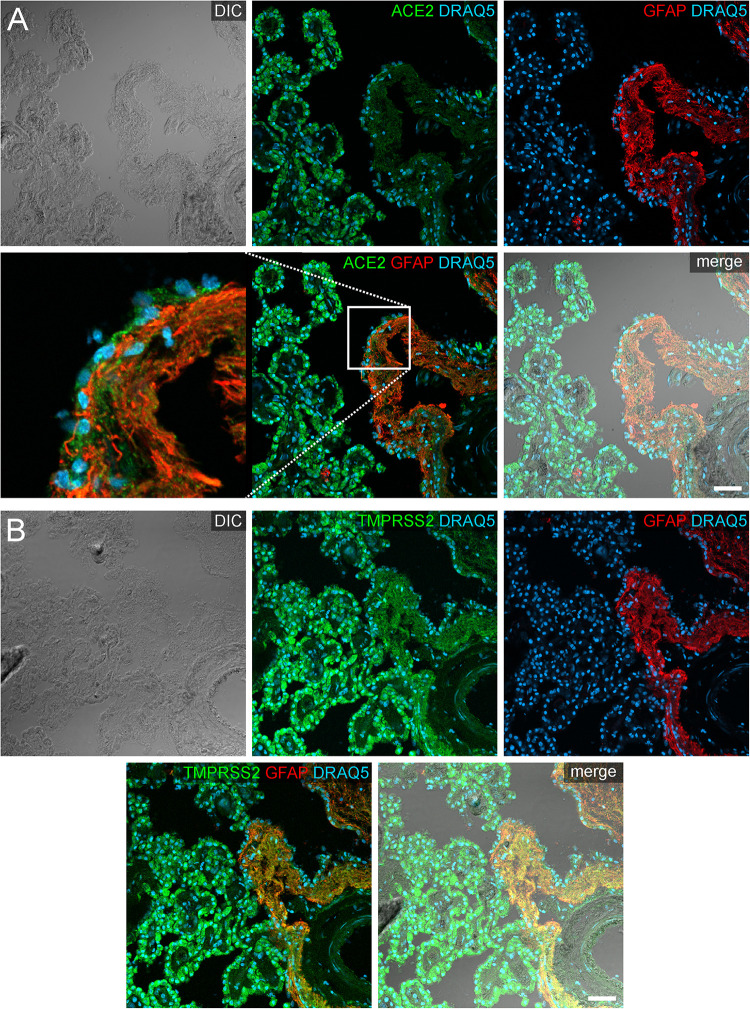
Expression of ACE2 and TMPRSS2 at the *taenia choroidea*. **(A)** Shows a DIC image of the interface between the human choroid plexus of the lateral ventricle and the brain parenchyma (i.e., *taenia choroidea*), as well as stainings for ACE2 (green), the glial marker GFAP (red), and the nuclear marker DRAQ5 (blue). The white rectangle indicates the location of the high power magnification micrograph showing ACE2 expression in ependymal cells at the ventricular surface. The immunoreactivity for ACE2 is much higher in plexus epithelial cells than in ependymal cells or astrocytic processes. **(B)** Shows a corresponding section as a DIC image and with stainings for TMPRSS2 (green), the glial marker GFAP (red), and the nuclear marker DRAQ5 (blue). Compared to the strong staining for TMPRSS2 in the plexus epithelial cells, astrocyte processes are weakly stained. All images are single optical sections (pinhole size 1 AU). Scale bars: 50 μm.

In addition to the plexus epithelium, we investigated attaching and accompanying tissues of the choroid plexus. The *Tela choroidea*, consisting of connective tissue and embedded blood vessels represents the stroma of the choroid plexus and is attached to the brain parenchyma at the so-called *Taenia*. At this *Taenia*, astroglial processes directly abut to the stromal connective tissue of the choroid plexus, and the plexus epithelium connects in a continuous sheet of cells to the ependyma lining the brain ventricles. The astrocytic processes identified by GFAP immunoreactivity showed an evenly distributed cytoplasmic staining for ACE2 that was less intense than in plexus epithelial cells (Figure 5). Interestingly, an equally weak ACE2 expression was found also in ependymal cells. Similarly, astrocyte processes were weakly positive for TMPRSS2. Ependymal cells, however, were largely devoid of TMPRSS2 immunoreactivity.

Within the brain parenchyma, only a subset of astrocytes were weakly positive for ACE2 and TMPRSS2 (Supplementary Figure S6). Occasionally, we detected both proteases located in some astrocytic endfeed at the blood-brain interface in the striatum and cortex. However, it is noteworthy that we were not able to confirm this finding for all capillaries in this brain area.

## Discussion

While the respiratory symptoms of COVID-19 are paramount in the treatment and current research, clinical evidence is accumulating that a SARS-CoV2 infection of the nervous system could aggravate the course of disease and arguably plays an important role in systemic disease progression (Li Y. C. et al., 2020; Li Z. et al., 2020). Although plenty of theories have been proposed on how and where corona virus particles could enter the nervous system (Briguglio et al., 2020), there is remarkably little histological and anatomical data available that would support any of these hypothesized routes of virus entry. A few studies have used gene expression analysis tools to identify mRNA expression of ACE2 and/or TMPRSS2 (Devaux et al., 2020; Zhang et al., 2020), yet these results must be interpreted with care since a post-transcriptional regulation of these genes by miRNAs has been reported (Devaux et al., 2020). In our study, we therefore evaluated the protein expression pattern of ACE2 and TMPRSS2, two proteinases involved in the cellular entry mechanism of SARS-CoV2, in two designated, often neglected, interfaces of the nervous system in the intestine and at the blood-CSF-barrier. Thus, we used small and large intestine resacted from pediatric patients (ranging from 3 months to 8 years and 5 months of age) and *post-mortem* specimens of the choroid plexus of the lateral ventricles.

In general, neuroinvasion can conceivably occur via a neural pathway (i.e., transferring the virus from neuron to neuron) or via body fluids (i.e., hematogenic, lymphogenic, or via the cerebrospinal fluid), as recently reviewed by Li Z. et al. (2020). Thus, an infection of the neuronal network of the ENS and successive transmission of SARS-CoV2 via the vagal, splanchnic, or spinal nerves into the CNS is therefore a potential route that is currently discussed (Briguglio et al., 2020). Interestingly, similar pathways have been proposed for other viral infections (Park et al., 2002) and for the gut-to-brain transport of misfolded α-synuclein in Parkinson’s disease (Holmqvist et al., 2014). Moreover, other corona virus infections, such as SARS or MERS, often exhibit gastrointestinal pathologies (Petrosillo et al., 2020), sometimes even preceding the respiratory symptoms (Mackay and Arden, 2015). Our results substantiate the hypothesis of an ENS-transmitted nervous system entry in that enteric neurons do express ACE2 and TMPRSS2, thereby meeting the histological prerequisites for such an infection. Interestingly, we found differences in the expression level of ACE2 in different enteric neurons, indicating that some neuronal subtypes and their respective networks might be more susceptible to SARS-CoV2 entry than others. This idea is also substantiated by our findings that ACE2 and especially TMPRSS2 expression was considerably stronger in ENS cells of the colon compared to the small intestine. However, we want to point out that ACE2 expression may also vary within one particular neuronal subtype and that immunohistological stainings do not allow precise quantification of protein expression levels. It is up to future research to verify our hypotheses and if applicable to identify more susceptible neuronal subtypes and the potential influence of their infection on the overall enteric function. It is noteworthy, however, that we only found expression of ACE2 and TMPRSS2 in the perikarya, but never in the neurites of the ENS, strongly indicating that an infection would need to take place within the ganglia and that a direct transmission from infected enterocytes to adjacent nerve endings appears unlikely. Moreover, our results were gained from pediatric specimens and future analyses should verify these findings in the ENS of adult and elderly patients.

Additionally, we found ACE2 and TMPRSS2 in glial cells of the ENS. Since enteric glial cells serve various functions, including immune-regulation and antigen presentation (Bassotti et al., 2007; da Silveira et al., 2011), an infection of these cells would potentially compromise a proper immune response, which in turn might contribute to a systemic spread of disease or a cytokine storm. Similar to this indirect effect of an ENS infection, other functions that are critically influenced by the ENS could suffer and cause additional symptoms. Thus, gut motility, intestinal blood flow, or the epithelial barrier could become dysfunctional, in turn hampering important defense mechanisms of the host against COVID-19 and other opportunistic superinfections (Briguglio et al., 2020). Moreover, recent reports link a higher incidence of glucose intolerance to corona virus infections (Chee et al., 2020; Rubino et al., 2020), in which ENS dysfunction arguably does play a role.

We also focused on histological evidence for a neuroinvasion of SARS-CoV2 via the choroid plexus. Viral agents have repeatedly shown to reach the CSF, often using the ensheathing cells of the olfactory nerve as an entry route (reviewed recently by Briguglio et al., 2020). Here, we evaluated the choroid plexus as an alternative route for CSF or brain entry. Since the endothelium of the blood vessels within the *Tela choroidea* are fenestrated, the epithelium of the choroid plexus represents the major element of the blood-CSF barrier (Wolburg and Mack, 2014; Pfeiffer et al., 2017). Indeed, a previous report suggests that ACE2 is expressed by the brush-border of the plexus epithelium; however, these results relied on enzyme activity in the brush border without any further histo-morphological correlation and were performed in sheep (Marshall et al., 2013). Thus, two entry routes into the choroid plexus epithelium appear possible, one apically via the CSF and one via the fenestrated blood vessels. We now found that human plexus epithelial cells express both ACE2 and TMPRSS2 on protein level and therefore are potential targets of a SARS-CoV2 infection. Intriguingly, our results show that both of these proteinases are mainly located in granules within the cytoplasm, with only occasional localization at the apical or basolateral cell surface. As this conceivably would have influence on the efficacy of viral entry, future research needs to elucidate whether a regulatory mechanism exists in order to control a translocation of ACE2 and TMPRSS2 from intracellular vesicle stores to the cell surface. Moreover, Dahm et al. (2016) recently reviewed possible entry mechanism used by viruses crossing the blood-brain-barrier. However, our weak stainings for ACE2 and TMPRSS2 in the brain parenchyma do not provide convincing evidence for this notion. This does not exclude the possibility of an ACE2-independent entry into the brain shuttled by immune cells (Dahm et al., 2016). Additionally, a neuronal transmission of SARS-CoV2 via the olfactory nerve appears unlikely since ACE2 is expressed in the olfactory epithelium by sustentacular cells, but not olfactory receptor neurons (Bilinska et al., 2020; Klingenstein et al., 2020).

It is also noteworthy that although viral nucleic acids have been found in the CSF of COVID-19 patients (Moriguchi et al., 2020), there still is very limited data to estimate the clinical relevance of SARS-CoV2 in the CSF. Just recently, however, Jacob et al. (2020) showed in their pre-publication manuscript that choroid plexus epithelial cells derived from human pluripotent stem cells *in vitro* were highly susceptible to SARS-CoV2 infections adding functional relevance to our morphological findings. Moreover, one should keep in mind that, similar to the ENS, an infection of the plexus epithelium could also have indirect effects on the proper functioning of CSF production as well as on water- and electrolyte homeostasis in the brain. Furthermore, an alternative entry route via the meningeal component of the *Tela choroidea* and a potential persistence of SARS-CoV2 in cells of the nervous system should be considered (Pfeiffer et al., 2017).

Taken together, our data provide fundamental evidence for potential alternative routes for the neuroinvasion of SARS-CoV2. The expression of ACE2 and TMPRSS2 in various cells of the ENS and the choroid plexus will need to be elucidated further in order to identify susceptible subpopulations. Also, the biological function of ACE2 and TMPRSS2 in the ENS and the choroid plexus remains elusive and a more detailed understanding of their physiological role might explain clinical phenomena. We critically want to point out that we did not provide any functional evidence for an actual infection of these cells by SARS-CoV2. Thus, functional transgenic animal models as well as post-mortem examinations of COVID-19 patients with neurological and/or gastroenterological symptoms will provide highly valuable insights.

## Data Availability Statement

The raw data supporting the conclusions of this article will be made available by the authors, without undue reservation.

## Ethics Statement

The studies involving human participants were reviewed and approved by Ethik-Kommission an der Medizinischen Fakultät der Eberhard-Karls-Universität und am Universitätsklinikum Tübingen. Written informed consent to participate in this study was provided by the participants’ legal guardian/next of kin.

## Author Contributions

FD and MS: acquisition of data, analysis and interpretation of data, drafting of the manuscript, and critical revision of the manuscript for important intellectual content. SK, MK, and AM: analysis and interpretation of data and critical revision of the manuscript for important intellectual content. SS: acquisition of patient material and critical revision of the manuscript for important intellectual content. AW: acquisition of data and critical revision of the manuscript for important intellectual content. BH: study concept and design, interpretation of data, and critical revision of the manuscript for important intellectual content. AFM and PN: study concept and design, acquisition of data, analysis and interpretation of data, drafting of the manuscript, critical revision of the manuscript for important intellectual content, and study supervision. All authors contributed to the article and approved the submitted version.

## Conflict of Interest

The authors declare that the research was conducted in the absence of any commercial or financial relationships that could be construed as a potential conflict of interest.

## Supplementary Material

FIGURE S1 Negative controls for antibody stainings. **(A,B)** Overviews of transversal sections of the human colon of two different patients treated with both secondary antibodies as a negative control and the nuclear stain DAPI (blue) for better orientation. The white rectangles indicate the location of the high power magnification micrographs on the right. Arrowheads point at enteric neurons in the submucous and myenteric plexus. **(C,D)** Overviews of sections through the human choroid plexus treated with secondary antibodies as a negative control and the nuclear stain DRAQ5 (blue) for better orientation. The microscope settings were the same as for the single channel stainings in Figure 4C and for double stainings in Figure 5D. **(A,B)** Are standard epifluorescence images; **(C,D)** are single optical sections (pinhole size 1 AU). Scale bars: **(A,B)** 500 μm; **(C,D)** 50 μm.

FIGURE S2 ACE2 expression in the intestinal epithelium. Overviews of the *Tunica muscosa* of the human small intestine **(A)** and colon **(B)** stained for ACE2 (red) and the nuclear stain DAPI (blue). The white rectangles indicate the location of the respective high power magnification insets. Especially the brush border of the small intestine exhibits a highly intense staining. All images are maximum intensity projections of optical slices by structured illumination. Scale bars: overviews 100 μm; insets 20 μm.

FIGURE S3 Variable expression levels of ACE2 in submucosal enteric neurons. **(A,B)** show maximum intensity projections of representative submucous ganglia of the human small intestine stained for ACE2 (red) and the nuclear stain DAPI (blue). Arrows point to enteric neurons with a weak expression of ACE2, arrowheads point to neurons with a highly intense ACE2 staining. Scale bars: 20 μm.

FIGURE S4 ACE2 expression in extraganglionic enteric glial cells. Shows type-III enteric glial cells (S100b in green) in the *Lamina propria mucosae* of the small **(A)** and large **(B)** intestine, not exhibiting any ACE2 immunoreactivity (red). **(C,D)** Depict extraganglionic glial cells in the *Tela submucosa*, most of which express ACE2. In **(D)**, enteric glial cells in close contact to blood vessels are shown, with ACE2 expressed especially by smooth muscle cells of the *Tunica media* and the glial cells, however, with varying intensity. **(E,F)** show type-IV enteric glial cells within the smooth musculature of the Tunica muscularis in the small **(E)** and large **(F)** intestine. ACE2 is expressed in these cell with the same intensity as in the surrounding muscle tissue. All images are maximum intensity projection of optical sections by structured illumination. Scale bars: 20 μm.

FIGURE S5 Differential subcellular localization of ACE2 in epithelial cells of the human choroid plexus. **(A)** Shows a DIC image of epithelial cells of the human choroid plexus. In **(B)** immunostainings for ACE2 (green) reveal two types of subcellular localization of the protease: in intracellular vesicles (arrow) or at the cell surface (arrowheads). Shown is a single optical section (pinhole size 1 AU). **(C)** The image is the merged image. Scale bars: 10 μm.

FIGURE S6 ACE2 and TMPRSS2 expression at the blood-brain barrier in the human striatum. **(A)** Shows a section of the human striatum immunostained for ACE2 (green) and GFAP (red). ACE2 can be detected in some astrocytic cell processes and occasionally surrounding vessels. In **(B)** immunostainings for TMPRSS2 (green) and GFAP (red) are depicted. TMPRSS2 is expressed in some astrocytic endfeed at the blood–brain interface. The asterisk indicates the lumen of a capillary. All images are single optical sections (pinhole size 1 AU). Scale bars: 20 μm.

## References

[B1] Bassotti G. Villanacci V. Antonelli E. Morelli A. Salerni B. (2007). Enteric glial cells: new players in gastrointestinal motility? *Lab Invest.* 87 628–632. 10.1038/labinvest.3700564 17483847

[B2] Bilinska K. Jakubowska P. Von Bartheld C. S. Butowt R. (2020). Expression of the SARS-CoV-2 entry proteins, ACE2 and TMPRSS2, in cells of the olfactory epithelium: identification of cell types and trends with age. *ACS Chem. Neurosci.* 11 1555–1562. 10.1021/acschemneuro.0c00210 32379417 PMC7241737

[B3] Bösel J. Berlit P. (2020). Neurological effects of COVID-19. *DGNeurologie* 3 277–284. 10.1007/s42451-020-00191-9.)

[B4] Briguglio M. Bona A. Porta M. Dell’Osso B. Pregliasco F. E. Banfi G. (2020). Disentangling the hypothesis of host dysosmia and SARS-CoV-2: the bait symptom that hides neglected neurophysiological routes. *Front. Physiol.* 11:671. 10.3389/fphys.2020.00671 PMC7292028 32581854

[B5] Brun P. Giron M. C. Zoppellaro C. Bin A. Porzionato A. De Caro R. (2010). Herpes simplex virus type 1 infection of the rat enteric nervous system evokes small-bowel neuromuscular abnormalities. *Gastroenterology* 138 1790–1801. 10.1053/j.gastro.2010.01.036 20102717

[B6] Cataldi M. Pignataro G. Taglialatela M. (2020). Neurobiology of coronaviruses: potential relevance for COVID-19. *Neurobiol. Dis.* 143:105007. 10.1016/j.nbd.2020.105007 32622086 PMC7329662

[B7] Chee Y. J. Ng S. J. H. Yeoh E. (2020). Diabetic ketoacidosis precipitated by Covid-19 in a patient with newly diagnosed diabetes mellitus. *Diabetes. Res. Clin. Pract.* 164:108166. 10.1016/j.diabres.2020.108166 32339533 PMC7194589

[B8] da Silveira A. B. de Oliveira E. C. Neto S. G. Luquetti A. O. Fujiwara R. T. Oliveira R. C. (2011). Enteroglial cells act as antigen-presenting cells in chagasic megacolon. *Hum. Pathol.* 42 522–532. 21208643 10.1016/j.humpath.2010.06.016

[B9] Dahm T. Rudolph H. Schwerk C. Schroten H. Tenenbaum T. (2016). Neuroinvasion and inflammation in viral central nervous system infections. *Mediators Inflamm.* 2016:8562805. 10.1155/2016/8562805 PMC4897715 27313404

[B10] De Felice F. G. Tovar-Moll F. Moll J. Munoz D. P. Ferreira S. T. (2020). Severe acute respiratory syndrome coronavirus 2 (SARS-CoV-2) and the central nervous system. *Trends Neurosci.* 43 355–357. 32359765 10.1016/j.tins.2020.04.004 PMC7172664

[B11] Devaux C. A. Rolain J. M. Raoult D. (2020). ACE2 receptor polymorphism: susceptibility to SARS-CoV-2, hypertension, multi-organ failure, and COVID-19 disease outcome. *J. Microbiol. Immunol. Infect.* 53 425–435. 10.1016/j.jmii.2020.04.015 32414646 PMC7201239

[B12] Esposito G. Pesce M. Seguella L. Sanseverino W. Lu J. Sarnelli G. (2020). Can the enteric nervous system be an alternative entrance door in SARS-CoV2 neuroinvasion? *Brain Behav. Immun.* 87 93–94. 10.1016/j.bbi.2020.04.060 32335192 PMC7179488

[B13] Hamming I. Timens W. Bulthuis M. L. Lely A. T. Navis G. van Goor H. (2004). Tissue distribution of ACE2 protein, the functional receptor for SARS coronavirus. A first step in understanding SARS pathogenesis. *J. Pathol.* 203 631–637. 10.1002/path.1570 15141377 PMC7167720

[B14] Hoffmann M. Kleine-Weber H. Schroeder S. Kruger N. Herrler T. Erichsen S. (2020). SARS-CoV-2 cell entry depends on ACE2 and TMPRSS2 and is blocked by a clinically proven protease inhibitor. *Cell* 181 271.e8–280.e8. 32142651 10.1016/j.cell.2020.02.052 PMC7102627

[B15] Holmqvist S. Chutna O. Bousset L. Aldrin-Kirk P. Li W. Björklund T. (2014). Direct evidence of Parkinson pathology spread from the gastrointestinal tract to the brain in rats. *Acta Neuropathol.* 128 805–820. 10.1007/s00401-014-1343-6 25296989

[B16] Hung E. C. Chim S. S. Chan P. K. Tong Y. K. Ng E. K. Chiu R. W. (2003). Detection of SARS coronavirus RNA in the cerebrospinal fluid of a patient with severe acute respiratory syndrome. *Clin. Chem.* 49 2108–2109. 14633896 10.1373/clinchem.2003.025437 PMC7108123

[B17] Jacob F. Pather S. R. Huang W.-K. Wong S. Z. H. Zhou H. Zhang F. (2020). Human pluripotent stem cell-derived neural cells and brain organoids reveal SARS-CoV-2 Neurotropism. *bioRxiv* [Preprint]. 10.1101/2020.07.28.225151 33010822 PMC7505550

[B18] Khoury-Hanold W. Yordy B. Kong P. Kong Y. Ge W. Szigeti-Buck K. (2016). Viral spread to enteric neurons links genital HSV-1 infection to toxic megacolon and lethality. *Cell Host Microbe* 19 788–799. 10.1016/j.chom.2016.05.008 27281569 PMC4902295

[B19] Klingenstein M. Klingenstein S. Neckel P. H. Mack A. F. Wagner A. Kleger A. (2020). Evidence of SARS-CoV2 entry protein ACE2 in the human nose and olfactory bulb. *bioRxiv* [Preprint]. 10.1159/000513040 PMC7900466 33486479

[B20] Leonardi M. Padovani A. McArthur J. C. (2020). Neurological manifestations associated with COVID-19: a review and a call for action. *J. Neurol.* 267 1573–1576. 10.1007/s00415-020-09896-z 32436101 PMC7238392

[B21] Li Y. C. Bai W. Z. Hashikawa T. (2020). The neuroinvasive potential of SARS-CoV2 may play a role in the respiratory failure of COVID-19 patients. *J. Med. Virol.* 92 552–555. 10.1002/jmv.25728 32104915 PMC7228394

[B22] Li Z. Liu T. Yang N. Han D. Mi X. Li Y. (2020). Neurological manifestations of patients with COVID-19: potential routes of SARS-CoV-2 neuroinvasion from the periphery to the brain. *Front. Med.* 1–9. 10.1007/s11684-020-0786-5 32367431 PMC7197033

[B23] Mackay I. M. Arden K. E. (2015). MERS coronavirus: diagnostics, epidemiology and transmission. *Virol. J.* 12:222. 10.1186/s12985-015-0439-5 PMC4687373 26695637

[B24] Marshall A. C. Shaltout H. A. Pirro N. T. Rose J. C. Diz D. I. Chappell M. C. (2013). Antenatal betamethasone exposure is associated with lower ANG-(1-7) and increased ACE in the CSF of adult sheep. *Am. J. Physiol. Regul. Integr. Comp. Physiol.* 305 R679–R688. 23948771 10.1152/ajpregu.00321.2013 PMC3798802

[B25] Moriguchi T. Harii N. Goto J. Harada D. Sugawara H. Takamino J. (2020). A first case of meningitis/encephalitis associated with SARS-Coronavirus-2. *Int. J. Infect. Dis.* 94 55–58. 32251791 10.1016/j.ijid.2020.03.062 PMC7195378

[B26] Park C. H. Ishinaka M. Takada A. Kida H. Kimura T. Ochiai K. (2002). The invasion routes of neurovirulent A/Hong Kong/483/97 (H5N1) influenza virus into the central nervous system after respiratory infection in mice. *Arch. Virol.* 147 1425–1436. 10.1007/s00705-001-0750-x 12111416

[B27] Petrosillo N. Viceconte G. Ergonul O. Ippolito G. Petersen E. (2020). COVID-19, SARS and MERS: are they closely related? *Clin. Microbiol. Infect.* 26 729–734. 10.1016/j.cmi.2020.03.026 32234451 PMC7176926

[B28] Pfeiffer F. Mack A. F. Wolburg H. (2017). “Topological aspects of the blood–brain and blood–cerebrospinal fluid barriers and their relevance in inflammation,” in *The Blood Brain Barrier and Inflammation*, eds Lyck R. Enzmann G. (Cham: Springer International Publishing), 23–48. 10.1007/978-3-319-45514-3_2

[B29] Qiu Y. Zhao Y. B. Wang Q. Li J. Y. Zhou Z. J. Liao C. H. (2020). Predicting the angiotensin converting enzyme 2 (ACE2) utilizing capability as the receptor of SARS-CoV-2. *Microbes Infect.* 22 221–225. 10.1016/j.micinf.2020.03.003 32199943 PMC7156207

[B30] Rubino F. Amiel S. A. Zimmet P. Alberti G. Bornstein S. Eckel R. H. (2020). New-Onset Diabetes in Covid-19. *N. Engl. J. Med.* 383, 789–790. 32530585 10.1056/NEJMc2018688 PMC7304415

[B31] Sun P. Qie S. Liu Z. Ren J. Li K. Xi J. (2020). Clinical characteristics of hospitalized patients with SARS-CoV-2 infection: a single arm meta-analysis. *J. Med. Virol.* 92 612–617. 10.1002/jmv.25735 32108351 PMC7228255

[B32] Wang D. Hu B. Hu C. Zhu F. Liu X. Zhang J. (2020). Clinical characteristics of 138 hospitalized patients with 2019 novel coronavirus-infected pneumonia in wuhan, China. *JAMA* 323 1061–1069. 10.1001/jama.2020.1585 32031570 PMC7042881

[B33] WHO (2020). *Coronavirus Disease (COVID-19) Situation Report – 209.* Geneva: World Health Organization.

[B34] Wolburg H. Mack A. F. (2014). Comment on the topology of mammalian blood–cerebrospinal fluid barrier. *Neurol. Psychiatry Brain Res.* 20 70–72. 10.1016/j.npbr.2014.10.004

[B35] Yan R. Zhang Y. Li Y. Xia L. Guo Y. Zhou Q. (2020). Structural basis for the recognition of SARS-CoV-2 by full-length human ACE2. *Science* 367 1444–1448. 10.1126/science.abb2762 32132184 PMC7164635

[B36] Yoo B. B. Mazmanian S. K. (2017). The enteric network: interactions between the immune and nervous systems of the gut. *Immunity* 46 910–926. 10.1016/j.immuni.2017.05.011 28636959 PMC5551410

[B37] Zhang H. Kang Z. Gong H. Xu D. Wang J. Li Z. (2020). Digestive system is a potential route of COVID-19: an analysis of single-cell coexpression pattern of key proteins in viral entry process. *Gut* 69 1010–1018. 10.1136/gutjnl-2020-320953

[B38] Zhou P. Yang X. L. Wang X. G. Hu B. Zhang L. Zhang W. (2020). A pneumonia outbreak associated with a new coronavirus of probable bat origin. *Nature* 579 270–273. 10.1038/s41586-020-2012-7 32015507 PMC7095418

[B39] Ziegler C. G. K. Allon S. J. Nyquist S. K. Mbano I. M. Miao V. N. Tzouanas C. N. (2020). SARS-CoV-2 receptor ACE2 is an interferon-stimulated gene in human airway epithelial cells and is detected in specific cell subsets across tissues. *Cell* 181 1016.e19–1035.e19. 32413319 10.1016/j.cell.2020.04.035 PMC7252096

