# Supplementary figures and images for: Histological Evidence for the Enteric Nervous System and the Choroid Plexus as Alternative Routes of Neuroinvasion by SARS-CoV2
Source: Front Neuroanat. 2020 Oct 6;14:596439. doi: 10.3389/fnana.2020.596439 (PMC7573115; doi:10.3389/fnana.2020.596439)

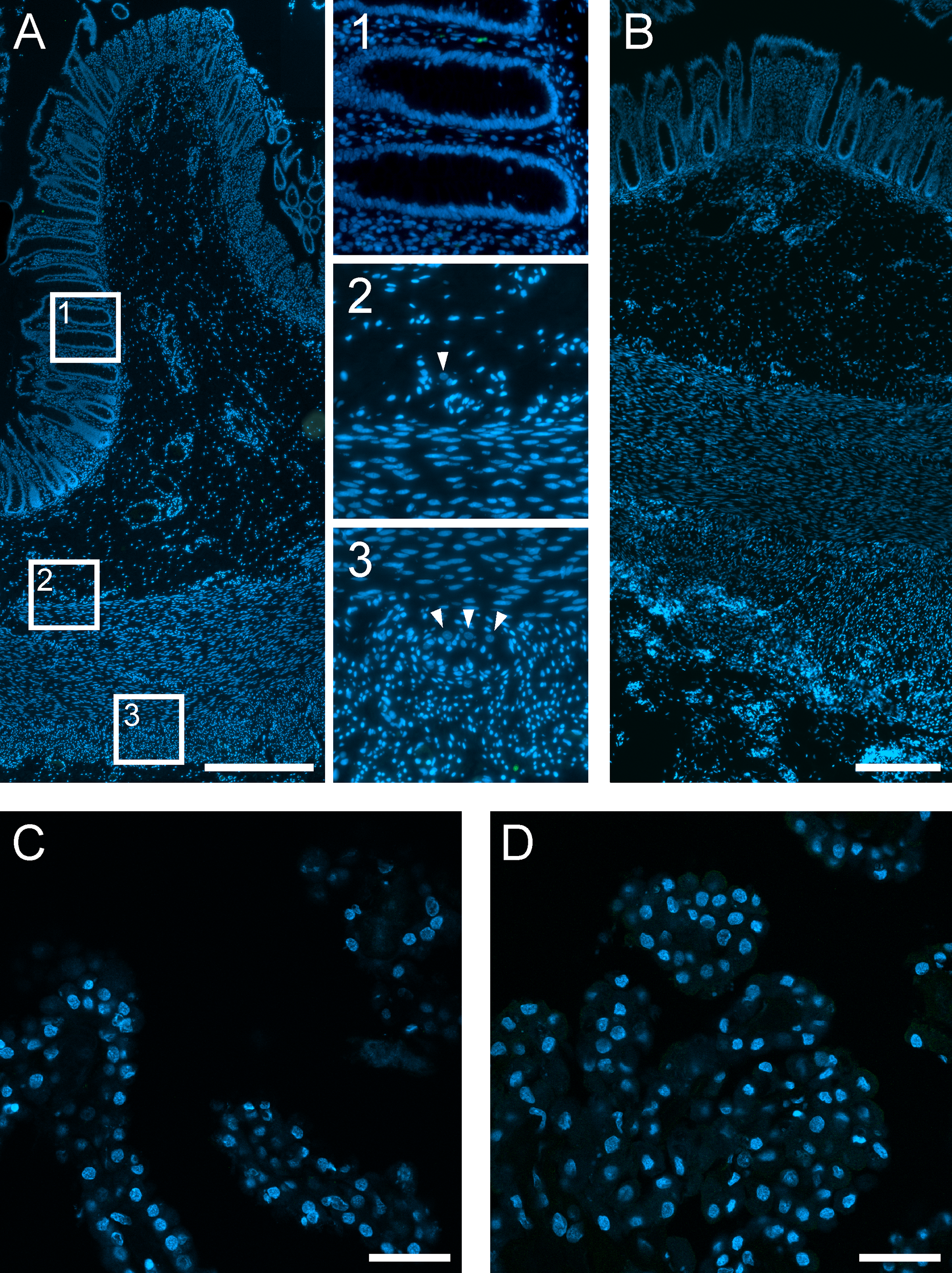

Supplement: FIGURE S1 — Negative controls for antibody stainings. (A,B) Overviews of transversal sections of the human colon of two different patients treated with both secondary antibodies as a negative control and the nuclear stain DAPI (blue) for better orientation. The white rectangles indicate the location of the high power magnification micrographs on the right. Arrowheads point at enteric neurons in the submucous and myenteric plexus. (C,D) Overviews of sections through the human choroid plexus treated with secondary antibodies as a negative control and the nuclear stain DRAQ5 (blue) for better orientation. The microscope settings were the same as for the single channel stainings in Figure 4C and for double stainings in Figure 5D. (A,B) Are standard epifluorescence images; (C,D) are single optical sections (pinhole size 1 AU). Scale bars: (A,B) 500 μm; (C,D) 50 μm. [file Image_1.TIF]

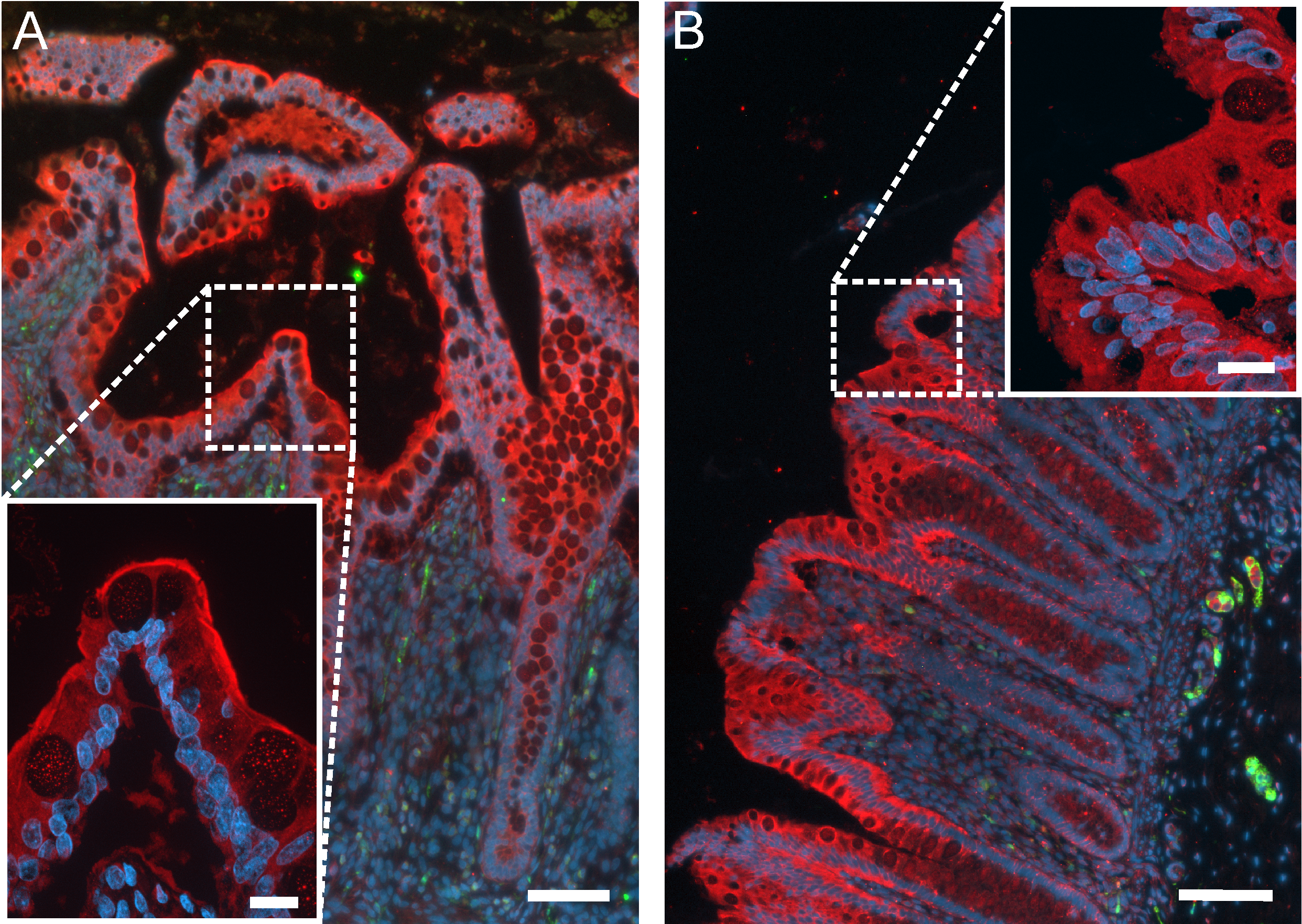

Supplement: FIGURE S2 — ACE2 expression in the intestinal epithelium. Overviews of the Tunica muscosa of the human small intestine (A) and colon (B) stained for ACE2 (red) and the nuclear stain DAPI (blue). The white rectangles indicate the location of the respective high power magnification insets. Especially the brush border of the small intestine exhibits a highly intense staining. All images are maximum intensity projections of optical slices by structured illumination. Scale bars: overviews 100 μm; insets 20 μm. [file Image_2.TIF]

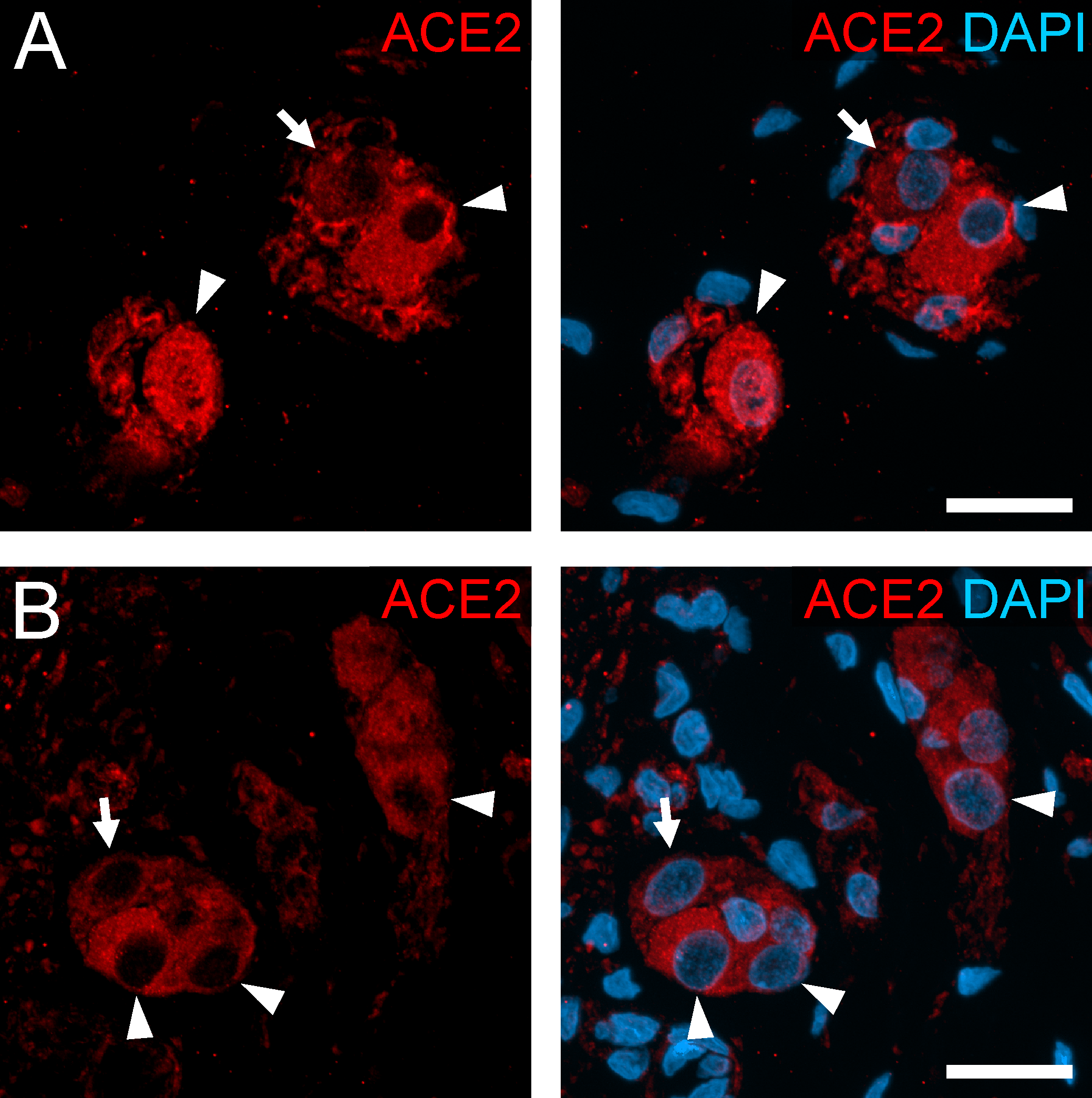

Supplement: FIGURE S3 — Variable expression levels of ACE2 in submucosal enteric neurons. (A,B) show maximum intensity projections of representative submucous ganglia of the human small intestine stained for ACE2 (red) and the nuclear stain DAPI (blue). Arrows point to enteric neurons with a weak expression of ACE2, arrowheads point to neurons with a highly intense ACE2 staining. Scale bars: 20 μm. [file Image_3.TIF]

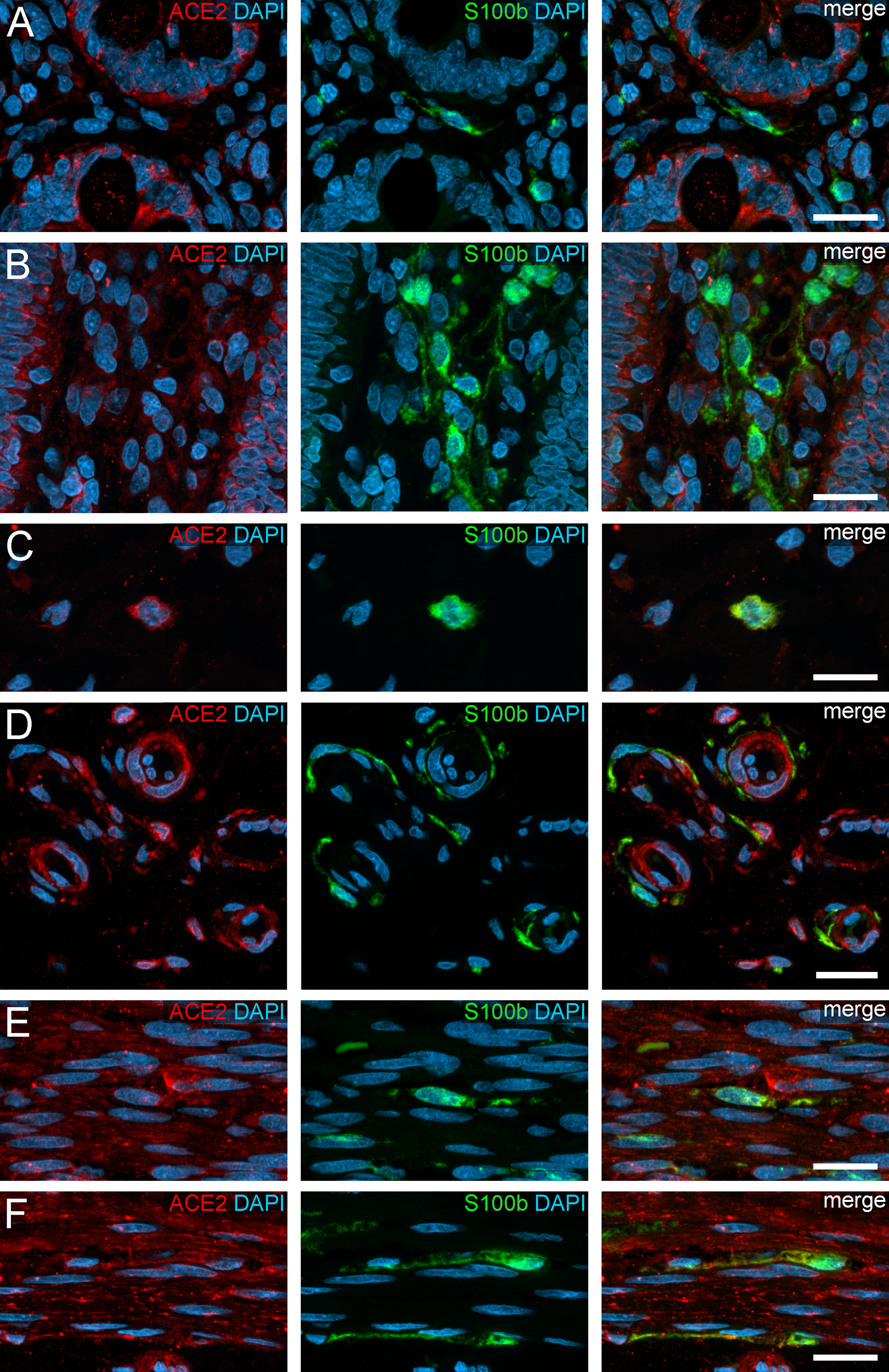

Supplement: FIGURE S4 — ACE2 expression in extraganglionic enteric glial cells. Shows type-III enteric glial cells (S100b in green) in the Lamina propria mucosae of the small (A) and large (B) intestine, not exhibiting any ACE2 immunoreactivity (red). (C,D) Depict extraganglionic glial cells in the Tela submucosa, most of which express ACE2. In (D), enteric glial cells in close contact to blood vessels are shown, with ACE2 expressed especially by smooth muscle cells of the Tunica media and the glial cells, however, with varying intensity. (E,F) show type-IV enteric glial cells within the smooth musculature of the Tunica muscularis in the small (E) and large (F) intestine. ACE2 is expressed in these cell with the same intensity as in the surrounding muscle tissue. All images are maximum intensity projection of optical sections by structured illumination. Scale bars: 20 μm. [file Image_4.TIF]

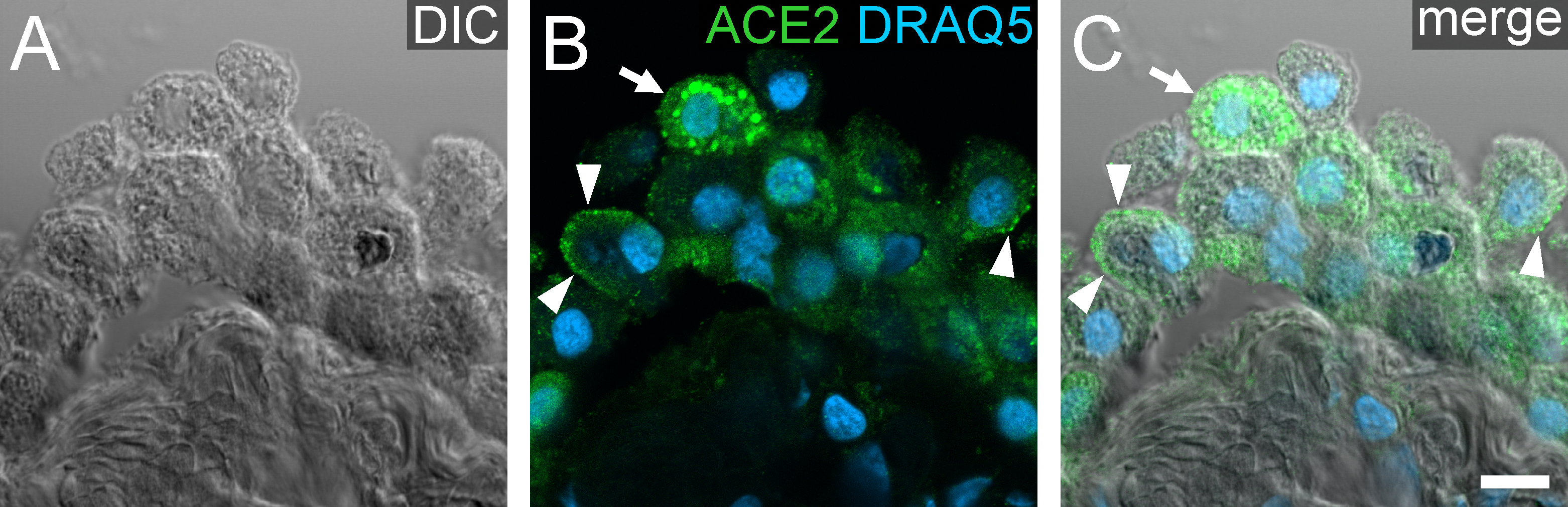

Supplement: FIGURE S5 — Differential subcellular localization of ACE2 in epithelial cells of the human choroid plexus. (A) Shows a DIC image of epithelial cells of the human choroid plexus. In (B) immunostainings for ACE2 (green) reveal two types of subcellular localization of the protease: in intracellular vesicles (arrow) or at the cell surface (arrowheads). Shown is a single optical section (pinhole size 1 AU). (C) The image is the merged image. Scale bars: 10 μm. [file Image_5.TIF]

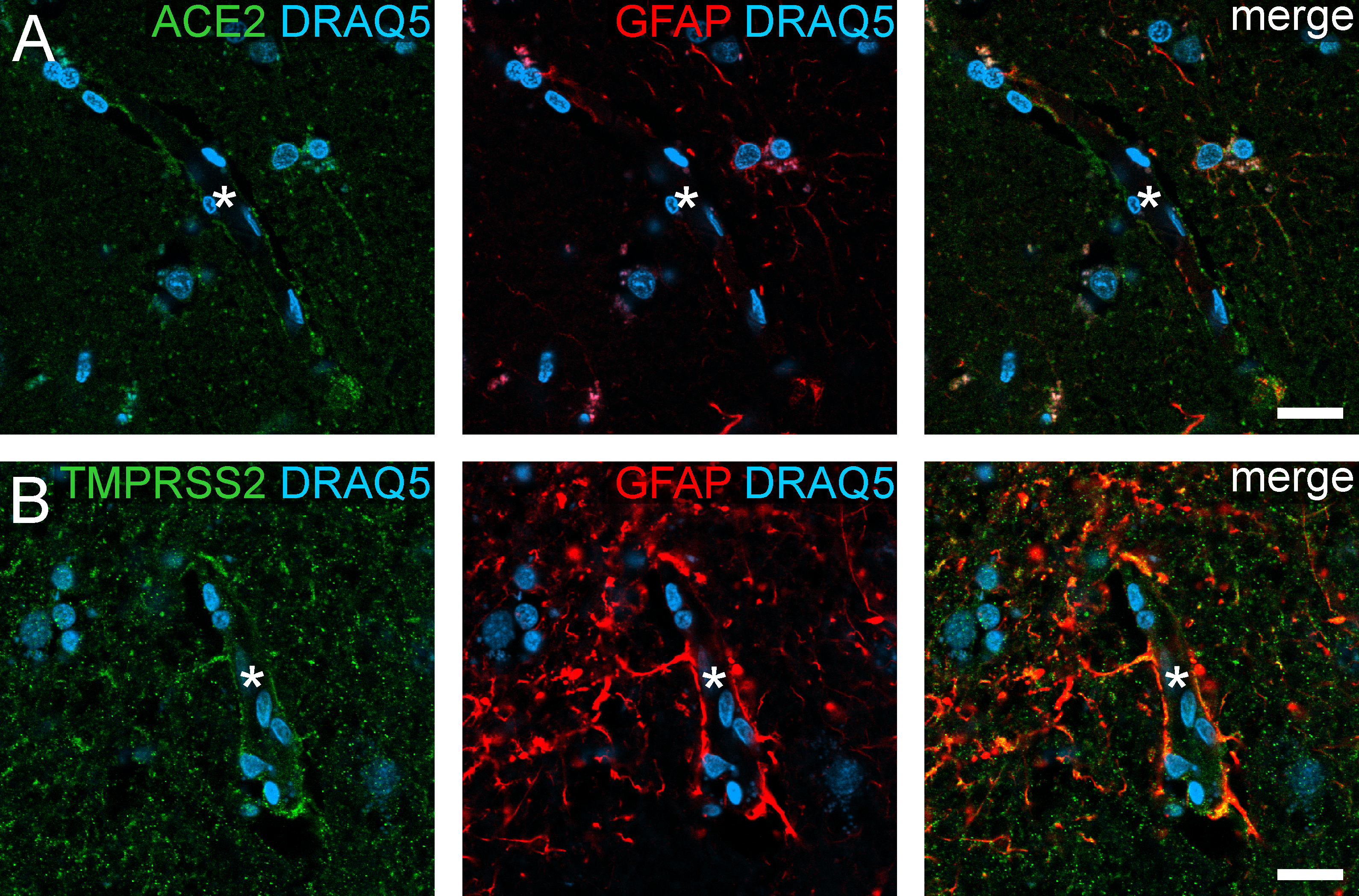

Supplement: FIGURE S6 — ACE2 and TMPRSS2 expression at the blood-brain barrier in the human striatum. (A) Shows a section of the human striatum immunostained for ACE2 (green) and GFAP (red). ACE2 can be detected in some astrocytic cell processes and occasionally surrounding vessels. In (B) immunostainings for TMPRSS2 (green) and GFAP (red) are depicted. TMPRSS2 is expressed in some astrocytic endfeed at the blood–brain interface. The asterisk indicates the lumen of a capillary. All images are single optical sections (pinhole size 1 AU). Scale bars: 20 μm. [file Image_6.TIF]
