# Supplementary material for: Histological Evidence for the Enteric Nervous System and the Choroid Plexus as Alternative Routes of Neuroinvasion by SARS-CoV2
Source: Front Neuroanat. 2020 Oct 6;14:596439. doi: 10.3389/fnana.2020.596439 (PMC7573115; doi:10.3389/fnana.2020.596439)
Supplement: Supplementary file 7 [file Table_1.DOCX]

**Supplementary tables**

**supplementary table 1:** intestinal resectates used in this study

| **age** | **sex** | **diagnosis** | **gut region** |
| --- | --- | --- | --- |
| 28 month | female | [imperforate anus](https://www.dict.cc/englisch-deutsch/imperforate+anus.html) | transverse colon |
| 31 month | female | [imperforate anus](https://www.dict.cc/englisch-deutsch/imperforate+anus.html) | transverse colon |
| 12 month | female | [imperforate anus](https://www.dict.cc/englisch-deutsch/imperforate+anus.html) | transverse colon |
| 3 month | male | intestinal obstruction syndrome | ileum |
| 10 month | male | [imperforate anus](https://www.dict.cc/englisch-deutsch/imperforate+anus.html) | descending colon |
| 14 month | female | rhabdomyosarcoma | transverse colon |
| 12 month | female | imperforate anus | transverse colon |
| 3 month | female | [imperforate anus](https://www.dict.cc/englisch-deutsch/imperforate+anus.html) | duodenum |
| 8 years 5 months | female | short bowel syndrom | duodenum |

**supplementary table 2:** body donors used in this study

| **age** | **sex** | **post mortem interval** | **cause of death*** |
| --- | --- | --- | --- |
| 85 years | male | 11 h | kidney failure |
| 83 years | female | 15 h | pulmonary carcinoma |
| 89 years | male | 12 h | kidney failure |
| 91 years | female | 19 h | exsiccosis, GI bleeding |
| 93 years | male | 11 h | pneumonia^#^ |
| 75 years | female | 8 h | ischemic stroke |
| 74 years | female | 9 h | multi organ failure |
| 94 years | female | 11 h | cardiac arrest |

* cause of death as it was written in the official death certificate after external examination

^#^ COVID-19 negative

**supplementary table 3:** primary antibodies used in this study

| **epitope** | **host** | **dilution** | **manufacturer** |
| --- | --- | --- | --- |
| ACE2 | rabbit | 1:100 | Abcam, Cambridge, UK |
| TMPRSS2 | rabbit | 1:500 | Abcam, Cambridge, UK |
| HuC/D | mouse | 1:50 | Invitrogen, CA, USA |
| PGP9.5 | mouse | 1:300 | Biorad AbD Serotech, Oxford, UK |
| GFAP | mouse | 1:100 | Merck Millipore, MA, USA |
| S100b | mouse | 1:50 | Abcam, Cambridge, UK |

**supplementary table 4:** secondary antibodies used in this study

| **antibody** | **Host** | **Dilution** | **manufacturer** |
| --- | --- | --- | --- |
| α-ms Alexa 546 | goat | 1:400 | Invitrogen, CA, USA |
| α-rb Alexa 488 | goat | 1:400 | Invitrogen, CA, USA |
